# Crosstalk between Aryl Hydrocarbon Receptor and Glucocorticoid Receptor in Human Retinal Pigment Epithelial Cells

**DOI:** 10.1155/2017/5679517

**Published:** 2017-03-22

**Authors:** Hong Lan Jin, Yujin Choi, Kwang Won Jeong

**Affiliations:** Gachon Institute of Pharmaceutical Sciences, College of Pharmacy, Gachon University, 191 Hambakmoero, Yeonsu-gu, Incheon 21936, Republic of Korea

## Abstract

The aryl hydrocarbon receptor (AHR) is known to mediate the cellular reaction involved in processing environmental contaminants and, ultimately, preventing accumulation of unfavorable extra lipids and proteins. Glucocorticoid receptor (GR) mediates the expression of genes associated with anti-inflammatory properties. Because AHR and GR are closely related in lipid metabolic dysregulation and inflammation, we speculate that AHR and GR may play a crucial role in AMD pathogenesis and focus on their crosstalk in human retinal pigment epithelial cells (ARPE-19). However, how AHR and GR regulate each other's signaling pathways is still poorly understood. In this research, we demonstrate that GR attenuates AHR-mediated gene expression by inhibition of nuclear translocation of AHR mediated by TCDD. Chromatin immunoprecipitation analysis demonstrated that GR repress AHR recruitment and chromatin accessibility response to TCDD + Dex treatment leading to repression of AHR target genes. In contrast, AHR facilitates GR-mediated expression in ARPE-19. AHR increases GR recruitment on GRE of GR target genes. Coimmunoprecipitation assay revealed that AHR is associated with GR in ARPE-19 cells and the interaction is enhanced by the addition of TCDD and Dex. Taken together, these studies provide a molecular mechanism of crosstalk between AHR and GR in target gene expression in ARPE-19 cells.

## 1. Introduction

Age-related macular degeneration (AMD) is characterized by degeneration of the retina. It is a well-known cause of vision loss in those over 55 years of age in the Western population [1]. Physiological changes of AMD begin with an accumulation of extracellular deposit consisting of rich lipid and protein under the retinal pigment epithelial (RPE) layer and/or within Bruch's membrane [2]. These deposits mainly regarded as drusen lead to RPE dysfunction, apoptosis, and eventually degeneration [3, 4]. There are 2 types of AMD; one is “dry AMD” and the other is “wet/exudative AMD.” The distinctive feature of wet AMD is neovascularization through the RPE layer and the choroid [5]. Dry AMD takes up to 90% of the entire AMD patients [6]. It often shows RPE dysfunction and local hyperpigmentation or bleaching [5]. As it progresses, the vision gets gradually worse, and finally “geographic atrophy” leads to vision loss. There is no treatment or medication option for Dry AMD [7]. Moreover, the molecular understanding of RPE dysfunction and mechanism for extracellular deposit accumulation is still unknown.

Emerging evidence suggests that aryl hydrocarbon receptor (AHR) signaling pathway may play an important role in AMD development. AHR mediates the cellular reaction involved in processing environmental contaminants such as polycyclic aromatic hydrocarbons (PAH), constituents of cigarette smoke, exhaust fumes, and fine dust, ultimately preventing accumulation of unfavorable extra lipids and proteins [8]. When the ligand binds AHR, the PAS domain of the AHR and AHR nuclear translocator (Arnt) protein heterodimerizes in the cytoplasm. The AHR-Arnt heterodimer then facilitates entering the nuclear membrane and subsequent binding to xenobiotics-response element (XRE) in DNA. This binding promotes expression of genes encoding xenobiotics and drug-metabolizing enzymes, genes related with extracellular matrix metabolism, and genes associated with biosynthesis, ubiquitination, proteasomal degradation, and clearance of cholesterol [9–13]. Recent studies have demonstrated that the aged AHR^−/−^ mice showed common phenotypes of dry AMD-like subretinal accumulation and focal RPE atrophy [7]. Also, it has been reported that the protein level of AHR in human primary RPE cells is continuously decreased by age, while freshly isolated cells from healthy donor eyes or ARPE-19, a spontaneously arising human cell line, have a relatively high amount of AHR protein [14]. Taken together, AHR performs as an essential defense system related to possible dry AMD pathology.

Glucocorticoid receptor (GR) also becomes influential in dry AMD progression. GR is usually considered as a stress-related transcription factor. It principally mediates gene expressions associated with anti-inflammatory properties. Upon ligand binding, GR dimer binds to a glucocorticoid response element (GRE) and starts to regulate target gene transcription [15]. GR is also found at high protein levels in the ARPE-19 cell line or freshly isolated human RPE cells [14]. In AMD treatment, triamcinolone, one of synthetic glucocorticoids, is prescribed for wet-type AMD patients expected anti-inflammatory outcome [16], and a recent study showed that the corticosteroid fluocinolone can inhibit VEGF expression in a GR-dependent way [17]. The clinical outcome of synthetic steroid hormone in dry-type AMD is not conclusive yet.

Both AHR and GR are closely related in development, reproduction, immune system regulation, and stress responses [18]. Previous studies suggest the interaction between AHR- and GR-mediated transcription. TCDD amplify GR transactivation by dexamethasone (Dex) in HepG2 HO23 cells, HepG2, 293T, and HeLa cells using reporter gene assay [19, 20]. On the other hand, it has been shown that Dex treatment suppresses AHR-mediated target gene expression [19]. Most recently, cotreatment of TCDD and hydrocortisone, AHR and GR agonists, induced a synergistic effect in embryonic cleft palate [21, 22]. AHR and GR signaling pathways play a critical role in the development and progression of AMD. Moreover, AHR and GR transactivation has a point of contact in the expression of genes encoding proinflammatory cytokines or drug-metabolizing enzyme. Thus, it is required to understand the interrelated mechanism of AHR and GR in retinal pigmented epithelial cell. However, not only the existence of their crosstalk in human retinal pigment cells but also the underlying mechanism remains largely unknown.

In this present study, we investigated the crosstalk between AHR- and GR-mediated transcription in APRE-19. To gain the insight of underlying molecular mechanism of the crosstalk, the nuclear localization of AHR and GR has been measured. Chromatin immunoprecipitation analysis demonstrated altered AHR and GR recruitment on XRE or GRE region of their target gene. Additionally, FAIRE-qPCR analysis has been used to monitor chromatin accessibility at the enhancer region of target genes after cotreatment of each ligand. This result encourages us to understand the potential mechanism for the expression of GR or AHR target genes influenced by the GR-AHR interaction in ARPE-19 cells, and finally, it may provide the information for understanding AMD pathogenesis.

## 2. Materials and Methods

### 2.1. Plasmids

The plasmid pCDNA-hGR was used for protein expression in this study and has been described previously [23].

### 2.2. Cell Culture

ARPE-19 human retinal pigment cells purchased from ATCC were maintained in DMEM-F12 medium with 10% fetal bovine serum (FBS) at 37°C in an atmosphere containing 5% CO_2_.

### 2.3. Quantitative RT-PCR (RT-qPCR)

To monitor the expression of AHR target genes, ARPE-19 cells were pretreated with TCDD (10 nM) for 2 h before Dex (100 nM) was added. A total of 6 h after the TCDD treatment, total RNA was isolated. Cells treated with TCDD (10 nM) for 6 hours or Dex (100 nM) for 4 hours were used as control group. To monitor the expression of GR target genes, ARPE-19 cells were pretreated with Dex (10 nM) for 2 h before TCDD (30 nM) was added. 4 h after Dex treatment, total RNA was isolated with Trizol (Invitrogen, Carlsbad, CA, USA) and subjected to reverse transcription (RT) using an iScript cDNA synthesis kit (Bio-Rad Laboratories, Hercules, CA, USA) in a total volume of 20 *μ*L. A 2 *μ*L aliquot of RT product was used for qPCR analysis. The RT-qPCR primer sequences are listed in Table 1. Expression levels were normalized to *18S* rRNA levels.

### 2.4. Protein Interaction Assays and Immunoblotting

ARPE-19 cells were treated for 30 min to Dex (100 nM), TCDD (30 nM), or Dex (100 nM) + TCDD (30 nM). The cytoplasmic and nuclear proteins were obtained from 90% cultures. The cytoplasmic lysis buffer contained the following: 1 M HEPES, 1 M KCL, 500 mM EDTA, NP-40, 1 M DTT, and a protease inhibitor. Following the collection of cytoplasmic proteins, the nuclei were lysed with the buffer containing 1 M Tris-HCl, 5 M NaCl, 1 M MgCl_2_, 500 mM EDTA, glycerol, and a protease inhibitor. A 25–50 *μ*g portion of protein lysates was used for western blotting. For the coimmunoprecipitation assay, ARPE-19 cells were plated at 1.5 × 10^6^ cells per 10 cm dish and transiently transfected using Lipofectamine 2000 (Invitrogen, Carlsbad, CA, USA) and 5 *μ*g of the plasmid. At 72 h after transfection (GR overexpression), cells were treated with TCDD (30 nM), Dex (100 nM), or TCDD (30 nM) + Dex (100 nM) for 30 min. Then, cell extracts were prepared in 1.0 mL of RIPA buffer (50 mM Tris-HCl (pH 8.0), 150 mM NaCl, 1% NP-40, 1% sodium deoxycholate, 0.1% sodium dodecyl sulfate, and 2 mM EDTA). Immunoblotting was performed as described previously [24], using anti-GR and anti-AHR antibodies (Santa Cruz Biotechnology, Dallas, TX, USA).

### 2.5. Chromatin Immunoprecipitation Assay

Chromatin immunoprecipitation (ChIP) assays were performed according to previously described protocols [24]. Briefly, ARPE-19 cells were cultured for 3 days in phenol red-free Dulbecco's modified Eagle's medium supplemented with 5% charcoal-dextran-stripped FBS. For AHR target genes, ARPE-19 cells were treated with TCDD (30 nM), Dex (100 nM), or TCDD (30 nM) + Dex (100 nM) for 30 min. For GR target genes, ARPE-19 cells were pretreated with Dex (2.5 nM) for 5 min before TCDD (30 nM) was added. 15 min after Dex treatment, cells were cross-linked using formaldehyde. Cells were cross-linked using formaldehyde. Cell extracts were prepared from control and Dex-treated cells. Immunoprecipitation of sonicated chromatin solutions with anti-AHR or anti-GR (Santa Cruz Biotechnology, Dallas, TX, USA) antibodies was conducted by overnight incubation at 4°C. Cross-linking was reversed by heating, and immunoprecipitated DNA was purified by phenol-chloroform extraction and ethanol precipitation. The purified DNA was dissolved in 100 *μ*L of TE buffer (10 mM Tris-HCl (pH 8.0) and 1 mM EDTA) and analyzed by qPCR using the Roche LightCycler 480 II System with SYBR Green dye. Results shown are the mean and range of variation of duplicate PCR analyses performed on the same DNA sample. The ChIP primer sequences are listed in Table 1.

### 2.6. Formaldehyde-Assisted Isolation of Regulatory Elements (FAIRE)-qPCR

FAIRE-qPCR for AHR target genes was performed as previously described [25]. Briefly, ARPE-19 cells were treated with TCDD (30 nM), Dex (100 nM), or TCDD (30 nM) + Dex (100 nM) for 30 min. Data are normalized against non-cross-linked genomic DNA for each primer pair. Results shown are means and the range of variation of duplicate PCR reactions performed on the same DNA sample. Results were expressed as a percentage of input chromatin (input) and were derived from a single experiment that is representative of at least two independent experiments. Sequences of FAIRE-qPCR primers are the same as those used for ChIP-qPCR analysis.

## 3. Results

### 3.1. Crosstalk of AHR and GR-Mediated Expression in ARPE-19

**Figure 1:**
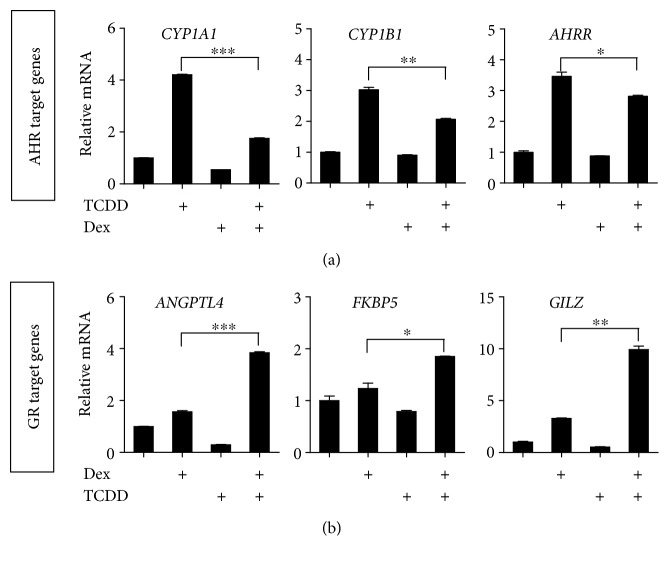
Crosstalk of AHR- and GR-mediated expression in ARPE-19. (a) ARPE-19 cells were treated with TCDD (10 nM), Dex (100 nM), or TCDD (10 nM) + Dex (100 nM). For the combination treatment, ARPE-19 cells were pretreated with TCDD for 2 h before Dex was added. 6 h after TCDD treatment, total RNA was isolated. The levels of *CYP1A1*, *CYP1B1*, and *AHRR* mRNA were normalized to the *18S* rRNA level. (b) ARPE-19 cells were pretreated with Dex (10 nM) for 2 h before TCDD (30 nM) was added. 4 h after Dex treatment, total RNA was isolated. The levels of *ANGPTL4*, *FKBP5*, and *GILZ* mRNA were normalized to the *18S* rRNA level. The results are presented as the mean ± SD of three independent experiments (*n* = 3). ^∗∗∗^*p* < 0.001, ^∗∗^*p* < 0.01, and ^∗^*p* < 0.05.

Previous studies have shown that AHR- and GR-mediated transcription is active in ARPE-19 cells with different target-gene specificity [26, 27], but there is no apparent study for their crosstalk in transcription process in ARPE-19. To determine whether AHR affects GR-mediated transcription or vice versa, ARPE-19 cells were cultured in DMEM/F-12 media, they were treated with TCDD and Dex alone or in combination, and then mRNA expression of AHR target genes (*CYP1A1*, *CYP1A2*, and *AHRR*) (Figure 1(a)) and GR target genes (*ANGPTL4*, *FKBP5*, and *GILZ*) (Figure 1(b)) were analyzed by quantitative real-time PCR. TCDD treatment for 6 hours resulted in a significant induction of AHR target genes (e.g., *CYP1A1*, *CYP1A2*, and *AHRR*). When ARPE-19 cells were treated with TCDD and Dex together, significant inhibition of expression of AHR target genes was observed compared to when cells were treated with TCDD only (Figure 1(a)). In contrast, the expression of GR target genes (e.g., *ANGPTL4*, *FKBP5*, and *GILZ*) induced by Dex was further enhanced by TCDD treatment in ARPE-19 cells (Figure 1(b)). TCDD alone did not show any effect on the expression of GR target genes. Similarly, Dex alone had no effect on expression of AHR target genes. These results suggest that crosstalk of AHR- and GR-mediated transcription is active in ARPE-19 cells with different target genes.

### 3.2. Dex Inhibits the Nuclear Translocation of AHR in ARPE-19

**Figure 2:**
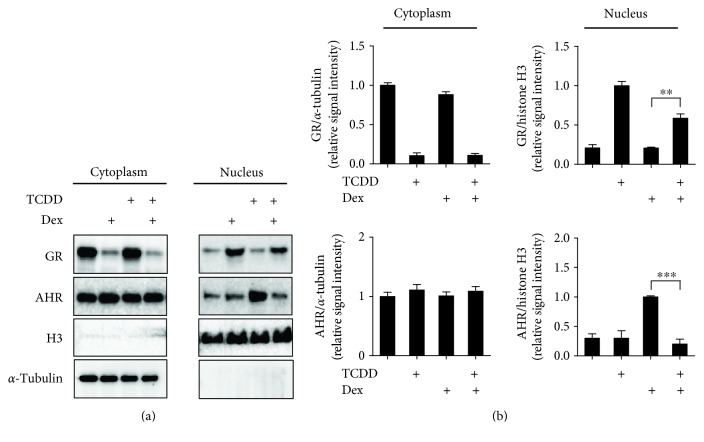
Dex inhibits the nuclear translocation of AHR in ARPE-19. (a) ARPE-19 cells were treated for 30 min to Dex (100 nM), TCDD (30 nM), or Dex (100 nM) + TCDD (30 nM). Western blot analysis of GR and AHR in cytoplasmic and nuclear fraction was performed. Histone H3 is used as a control for a nuclear fraction, and *α*-tubulin is used as a control for cytoplasmic fraction. (b) The relative protein levels of GR and AHR were quantified by scanning densitometry and normalized to *α*-tubulin (cytoplasmic fraction) and histone H3 (nuclear fraction), respectively. The images shown are representatives of three independent experiments that showed consistent results, and the relative protein values are expressed as mean ± SD for three independent experiments. ^∗∗∗^*p* < 0.001 and ^∗∗^*p* < 0.01.

To assess whether changes in mRNA levels reflect variations of AHR and GR protein stability or nuclear translocation, we quantified GR and AHR protein levels in cytoplasmic and nuclear fraction of ARPE-19 cells in the presence of Dex, TCDD, or Dex + TCDD (Figure 2). Histone H3 was used as a control for nuclear fraction, and *α*-tubulin was used as a control for cytoplasmic fraction. We observed AHR translocated to the nucleus after TCDD treatment and Dex-induced translocation of GR from the cytoplasm to the nucleus. While cotreated with TCDD + Dex, AHR translocation was significantly decreased. In contrast, GR translocation was also significantly reduced by TCDD + Dex, consistent with a previous report that Dex downregulated AHR protein level by AHR degradation in the presence of AHR ligand in JEG-3 cells [28]. Our results suggest that the reduction of AHR nuclear translocation by Dex treatment might be a critical mechanism of inhibition of AHR target gene expression by Dex treatment.

### 3.3. Decrease of AHR Recruitment to Its Target Genes by Dex

**Figure 3:**
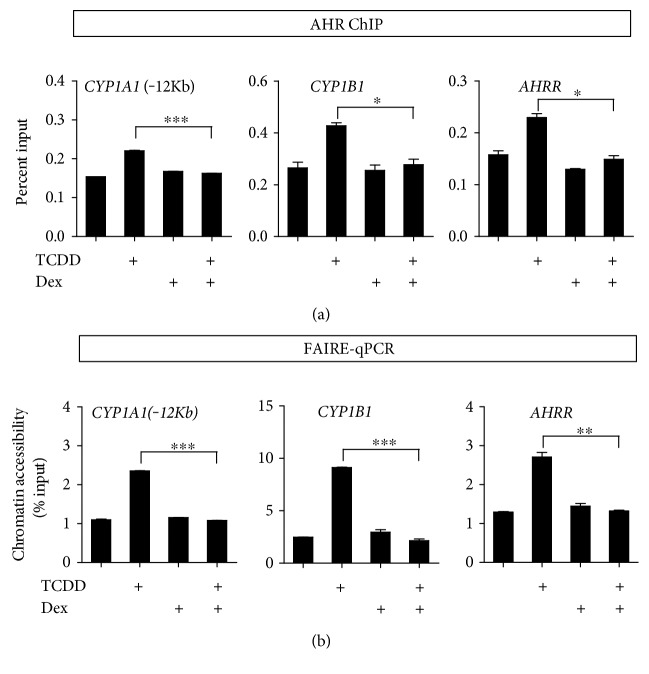
Decrease of AHR recruitment to its target genes by Dex. (a) AHR recruitment to AHR target genes. ARPE-19 cells were treated with TCDD (30 nM), Dex (100 nM), or TCDD (30 nM) + Dex (100 nM) for 30 min. Cells were cross-linked using formaldehyde and subjected to ChIP using antibodies against AHR. Immunoprecipitated DNA was quantified by qPCR using primers for *CYP1A1* (−12 kb), *CYP1B1*, and *AHRR*. (b) Chromatin accessibility at the AHR target genes was assessed by FAIRE-qPCR analysis by using chromatin samples prepared from ARPE-19 cells treated with TCDD (30 nM), Dex (100 nM), or TCDD (30 nM) + Dex (100 nM) for 30 min. Data are normalized against non-cross-linked genomic DNA for each primer pair. The results are presented as the mean ± SD of three independent experiments (*n* = 3). ^∗∗∗^*p* < 0.001, ^∗∗^*p* < 0.01, and ^∗^*p* < 0.05.

Since Dex treatment inhibited nuclear translocation of AHR, we tested whether the recruitment of AHR to this XRE is also affected by Dex treatment. First, we observed that TCDD treatment led to the recruitment of AHR to the target genes such as *CYP1A1*, *CYP1A2*, and *AHRR.* Dex treatment alone did not show any effect on AHR recruitment compared with vehicle control. However, the combination of TCDD and Dex inhibited AHR recruitment to all the three AHR target genes compared with TCDD treatment alone (Figure 3(a)). Given that GR repress AHR recruitment to AHR target genes, we used FAIRE-qPCR to investigate whether GR influences the chromatin accessibility of the AHR target genes of ARPE-19 cells. We performed formaldehyde-assisted isolation of regulatory elements (FAIRE) coupled with qPCR. ARPE-19 cells were treated with TCDD, Dex, or TCDD + Dex for 30 min. We observed the increased in the FAIRE signal after TCDD treatment at the AHR binding sites. However, a combination of TCDD and Dex dramatically decreased the TCDD-induced FAIRE signal of the enhancer or promoter regions of the AHR target genes (Figure 3(b)). These results suggest that the reduced nuclear translocation of AHR by Dex treatment affect the recruitment of AHR to its target genes and the formation of open chromatin structure, resulting in the decrease of transcription.

### 3.4. AHR Increases GR Recruitment on GR-Binding Sites of GR Target Genes

**Figure 4:**
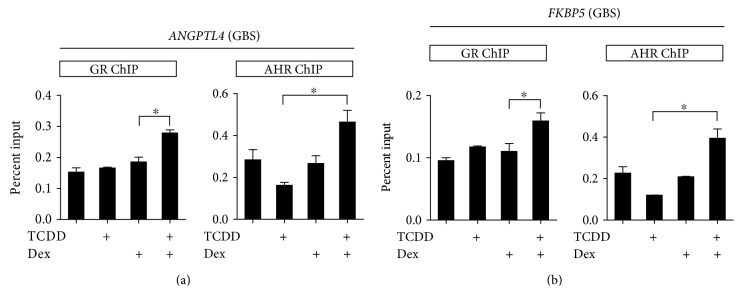
AHR increases GR recruitment on GBS of GR target genes. (a) and (b) ARPE-19 cells were treated with Dex (2.5 nM), TCDD (30 nM), or Dex (2.5 nM) + TCDD (30 nM). For the combination treatment, ARPE-19 cells were treated with Dex for 5 min before TCDD treatment. After TCDD treatment for 10 min, cells were cross-linked using formaldehyde and subjected to ChIP using antibodies against GR and AHR. Immunoprecipitated DNA was quantified by qPCR using primers for *ANGPTL4* (GBS) and *FKBP5* (GBS). The results are presented as the mean ± SD of three independent experiments (*n* = 3). ^∗^*p* < 0.05. GBS: GR-binding sites.

To elucidate the mechanism involved in AHR-mediated activation of GR-mediated transcription, we monitored the recruitment of AHR and GR at GR target genes (e.g., ANGPTL4 and FKBP5) in ARPE-19 cells. For better observation of TCDD effect on GR recruitment, we used a low concentration of Dex where a marginal increase of GR binding is observed. Thus, no significant increase in GR occupancy by Dex treatment was detected. However, cotreatment with TCDD caused a further increase of GR binding to GBSs (GR-binding sites) of *ANGPLT4* and *FKBP5* genes (Figure 4). ChIP assay using immunoprecipitation of AHR antibody showed that AHR recruitments were also increased in GBS both in *ANGPTL4* (Figure 4(a)) and *FKBP5* (Figure 4(a)) genes. These results suggest that the increased recruitment of GR and AHR by Dex + TCDD to GR target genes facilitates the activation of transcription.

### 3.5. The Interaction between AHR and GR Induced by TCDD and Dex

**Figure 5:**
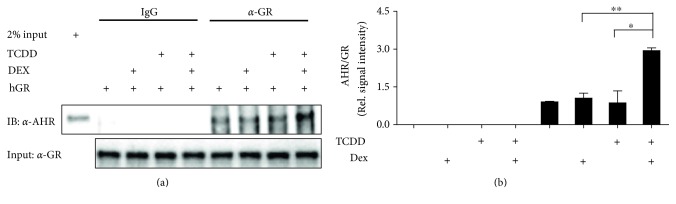
The interaction between AHR and GR induced by TCDD and Dex. A coimmunoprecipitation assay was carried out using ARPE-19 cells transfected with GR expression plasmid. At 72 h after transfection, cells were treated with TCDD (30 nM), Dex (100 nM), or TCDD (30 nM) + Dex (100 nM) for 30 min. Then, cell lysates were prepared in RIPA buffer. Coimmunoprecipitation assay was performed using normal mouse IgG or anti-GR antibody. Immunoblotting was performed using an anti-AHR antibody or anti-GR antibody. The relative protein levels of AHR were quantified by scanning densitometry and normalized to GR. The images shown are representatives of three independent experiments that showed consistent results, and the relative protein values are expressed as mean ± SD for three independent experiments. ^∗∗^*p* < 0.01 and ^∗^*p* < 0.05.

Given that the recruitment of GR to target gene is enhanced by TCDD, and AHR is also recruited to GR target gene in the presence of Dex and TCDD, we examined the possibility of a physical interaction between AHR and GR in ARPE-19 cells. We observed a weak interaction between AHR and GR. However, the interaction was enhanced by the addition of TCDD together with Dex (Figure 5), supporting the involvement of AHR to GR-mediated transcription in the presence of Dex + TCDD.

## 4. Discussion

Age-related macular degeneration is the leading cause of irreversible blindness and visual impairment. Physiological changes of AMD begin with an accumulation of the age pigment lipofuscin, on or within Bruch's membrane [29]. The human retinal pigment epithelium (RPE) is a monolayer of pigmented cells within the eye which plays a critical role in retinal physiology and the visual cycle [30]. The RPE cell layer forms the outer blood-retinal barrier called BRB. It has a fundamental role in supporting photoreceptors such as bring nutrients from the outer blood supply and dispose of metabolic products from subretinal space back to the blood [14]. Likewise, RPE cells are essential for keeping the vitality of neurosensory retina and the characteristic of AMD starts with the degeneration of RPE layer.

The aryl hydrocarbon receptor (AHR) is a nuclear receptor that regulates xenobiotic metabolism and an inhibitory regulator of lipid synthesis [31]. Recent studies demonstrated that AHR deficiency causes dysregulated cellular matrix metabolism and age-related macular degeneration-like pathology [32]. Moreover, AhR^−/−^ mice exhibit accumulation of RPE cell lipofuscin to decrease visual function and develop dry AMD-like pathology [32]. As such, glucocorticoid receptor (GR), a stress transcription factor, has shown to play a role in RPE physiology and AMD progression. Activation of GR by Dex-induced proliferation of cultured retinal pigment epithelial cells [33] and loss of GR in the retina result in the thinning of the inner nuclear layer of the peripheral retina in adult mice [34]. Because AHR and GR are closely related in AMD progression, we attempted to study AHR and GR crosstalk in ARPE-19 cells. The crosstalk of AHR with GR receptors has been reported in several cell lines; however, the existence of their crosstalk in human retinal pigmented cell (e.g., ARPE-19) and the underlying mechanism remain largely unknown.

In this study, we demonstrated that when ARPE-19 cells were treated with Dex and TCDD in combination, the expression of AHR target genes was decreased compared with that observed in the presence of TCDD only in ARPE-19 cells (Figure 1(a)). These results support previous studies showing that Dex suppressed BaP-induced AHR expression in human HepG2, HeLa, and Ho23 cells [35–37]. In contrast, several results have shown that the cotreatment with BaP and Dex caused a further weak increase in Dex-induced GR expression in HepG2 and HO23 cells [36, 20]. Similar results derived from ARPE-19 cells were obtained from our study. Dex-induced GR target gene expression was enhanced by TCDD treatment in ARPE-19 cells (Figure 1(b)). These results indicated that crosstalk of AHR- and GR-mediated transcription is active in ARPE-19 cells with different target genes.

Previous studies have demonstrated that AHR and GR proteins were decreased by cotreatment with TCDD + Dex compared with the basal level in HepG2 cells [36]. However, it was unclear whether the treatment of TCDD or Dex affects nuclear translocation of these nuclear receptors. In the present study, we examined the effect of TCDD, Dex, and their combinations on the translocation of AHR and GR from cytosol to nucleus. Endogenous GR decreased in the cytoplasm and increased in the nucleus after treatment with Dex, but the GR protein level slightly decreased in the nucleus by TCDD + Dex treatment. On the other hand, we demonstrated for the first time that treatment with TCDD + Dex decreased AHR nuclear translocation (Figure 2). These results suggested that TCDD and Dex in combination might play an important role in the recruitment of AHR and GR to their target genes. Indeed, the increase in AHR recruitment to the AHR target genes by TCDD treatment was remarkably inhibited by TCDD + Dex treatment. The FAIRE-qPCR results also support that TCDD and Dex in combination in ARPE-19 cells resulted in significant inhibition of chromatin accessibility of the AHR target genes (Figure 3). For GR target genes, the addition of Dex with TCDD enhanced the GR and AHR recruitment to the GBS (GR binding sites) of *ANGPTL4* (Figure 4(a)) and *FKBP5* (Figure 4(b)). This result indicated that the increase of GR target gene expression by Dex + TCDD is likely by the increased recruitment of GR and AHR to GR target genes, and these changes possibly facilitate the activation of transcription.

Additionally, we demonstrated that AHR is associated with GR in ARPE-19 cells and that coimmunoprecipitation of GR was increased by TCDD + Dex treatment (Figure 5). These results indicate that the interaction of AHR with GR is ligand dependent and suggest that this interaction plays an important role in the recruitment of AHR or GR to their target genes. Taken together, our results provide a transcriptional mechanism of aryl hydrocarbon receptor crosstalk with glucocorticoid receptor in human retinal pigment epithelial cells.

## Conflicts of Interest

The authors declare that there are no competing interests regarding the publication of this paper.

## Figures and Tables

**Table 1:** Primers used for RT-qPCR and ChIP experiments.

Gene	Forward primer sequence (5′ → 3′)	Reverse primer sequence (5′ → 3′)
*CYP1A1*	TGAACCCCAGGGTACAGAGA	GGCCTCCATATAGGGCAGAT
*CYP1B1*	AACGTACCGGCCACTATCAC	CCACGACCTGATCCAATTCT
*AHRR*	AGACTCCAGGACCCACAAAG	CATCCTCACTGTGCTTTCCC
*ANGPTL4*	TCCGCAGGGACAAGAACTG	TTGGAATGGCTGCAGGTGC
*FKBP5*	AGGCTGCAAGACTGCAGATC	CTTGCCCATTGCTTTATTGG
*GILZ*	AGATCGAACAGGCCATGGAT	TTACACCGCAGAACCACCAG
*18S*	GAGGATGAGGTGGAACGTGT	TCTTCAGTCGCTCCAGGTCT
*CYP1A1* (−12 kb)	AGTGGCTCACGCCAGTAATC	CGTGTTAGCCAGGATGGTCT
*CYP1B1* (XRE)	ATGACTGGAGCCGACTTTCC	GGCGAACTTTATCGGGTTG
*AHRR* (XRE)	CCCTGAGCCAAGGTGTGGAGC	TGCAGGCCTAGGGGGAACCAT
*ANGPTL4* GBS	ATGGGGAAAAGCTAATAGGGGAGG	TGCTCAGAAGGGAACGGGGTT
*FKBP* 5GBS	TGTGCCAGCCACATTCAGAACA	GTAACCACATCAAGCGAGCTG
